# A multidisciplinary team-oriented intervention to increase guideline recommended care for high-risk prostate cancer: A stepped-wedge cluster randomised implementation trial

**DOI:** 10.1186/s13012-018-0733-x

**Published:** 2018-03-12

**Authors:** Bernadette Brown, Jane Young, David P. Smith, Andrew B. Kneebone, Andrew J. Brooks, Sam Egger, Miranda Xhilaga, Amanda Dominello, Dianne L. O’Connell, Mary Haines

**Affiliations:** 1 Sax Institute, Haymarket, Australia; 2 School of Public Health, University of Sydney, Camperdown, Australia; 3 Cancer Research Division, Cancer Council NSW, Sydney, Australia; 4 Griffith Health Institute, Griffith University, Gold Coast, QLD Australia; 5 Department of Radiation Oncology, Royal North Shore Hospital, Sydney, Australia; 6 Northern Clinical School, University of Sydney, Camperdown, Australia; 7 NSW Agency for Clinical Innovation, Sydney, Australia; 8 Westmead Private Hospital, Westmead, Australia; 9 Westmead Clinical School, University of Sydney, Camperdown, Australia; 10 Prostate Cancer Foundation of Australia, Melbourne, Australia; 11 School of Medicine and Public Health, University of Newcastle, Callaghan, NSW Australia

## Abstract

**Background:**

This study assessed whether a theoretically conceptualised tailored intervention centred on multidisciplinary teams (MDTs) increased clinician referral behaviours in line with clinical practice guideline recommendations.

**Methods:**

Nine hospital Sites in New South Wales (NSW), Australia with a urological MDT and involvement in a state-wide urological clinical network participated in this pragmatic stepped wedge, cluster randomised implementation trial. Intervention strategies included flagging of high-risk patients by pathologists, clinical leadership, education, and audit and feedback of individuals’ and study Sites’ practices. The primary outcome was the proportion of patients referred to radiation oncology within 4 months after prostatectomy. Secondary outcomes were proportion of patients discussed at a MDT meeting within 4 months after surgery; proportion of patients who consulted a radiation oncologist within 6 months; and the proportion who commenced radiotherapy within 6 months. Urologists’ attitudes towards adjuvant radiotherapy were surveyed pre- and post-intervention. A process evaluation measured intervention fidelity, response to intervention components and contextual factors that impacted on implementation and sustainability.

**Results:**

Records for 1071 high-risk post-RP patients operated on by 37 urologists were reviewed: 505 control-phase; and 407 intervention-phase. The proportion of patients discussed at a MDT meeting increased from 17% in the control-phase to 59% in the intervention-phase (adjusted RR = 4.32; 95% CI [2.40 to 7.75]; *p* < 0·001). After adjustment, there was no significant difference in referral to radiation oncology (intervention 32% vs control 30%; adjusted RR = 1.06; 95% CI [0.74 to 1.51]; *p* = 0.879). Sites with the largest relative increases in the percentage of patients discussed also tended to have greater increases in referral (*p* = 0·001). In the intervention phase, urologists failed to provide referrals to more than half of patients whom the MDT had recommended for referral (78 of 140; 56%).

**Conclusions:**

The intervention resulted in significantly more patients being discussed by a MDT. However, the recommendations from MDTs were not uniformly recorded or followed. Although practice varied markedly between MDTs, the intervention did not result in a significant overall change in referral rates, probably reflecting a lack of change in urologists’ attitudes. Our results suggest that interventions focused on structures and processes that enable health system-level change, rather than those focused on individual-level change, are likely to have the greatest effect.

**Trial registration:**

Australian New Zealand Clinical Trials Registry (ANZCTR): ACTRN12611001251910). Registered 6 December 2011.

**Electronic supplementary material:**

The online version of this article (10.1186/s13012-018-0733-x) contains supplementary material, which is available to authorized users.

## Background

Discrepancies between research evidence and clinical practice remain one of the most persistent problems in the provision of high-quality health care. Clinical practice guidelines aim to inform clinical decision-making by providing summaries of recent, credible research evidence with recommendations for clinical practice. However, timely and effective implementation of guidelines into practice is inconsistent [1]. Hence, there is a need to evaluate interventions to determine how to most effectively make changes across the health system and promote timely implementation of guidelines.

Radical prostatectomy (RP) is the most frequently used treatment for locally advanced prostate cancer. Following RP, however, it is estimated that between 20% and 50% of men are at “high risk” of experiencing progression or recurrence [2, 3]. Three randomised controlled trials have demonstrated benefits in survival, recurrence and disease progression in RP patients with high-risk disease characteristics who receive adjuvant radiotherapy [4–6]. On the basis of this evidence, international clinical practice guidelines [7–9] recommend that after RP, men with one or more high-risk features (namely, extracapsular extension, seminal vesicle invasion or positive surgical margin) should be referred for consideration of adjuvant radiotherapy. Historically, data consistently show only approximately 10–20% of eligible men receive adjuvant radiotherapy in Australia [10–12] and other regions such as the USA and Canada [13–17].

**Fig. 1:**
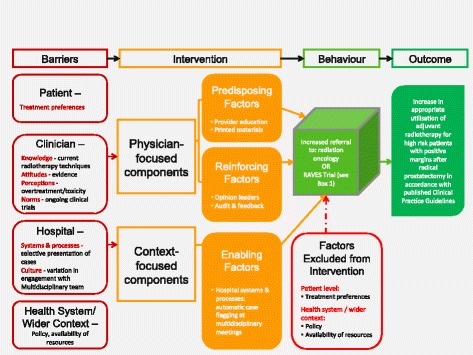
CLICC conceptual program logic framework

The Clinician-Led Improvement in Cancer Care (CLICC) implementation trial [18] evaluated a multi-faceted intervention designed to increase urologists’ referrals to radiation oncology in line with guideline recommended care. Implemented in New South Wales (NSW), Australia, the trial was embedded in clinical practice through the NSW Agency for Clinical Innovation Urology Clinical Network. Described fully in the protocol [18] and outlined below, the CLICC intervention was informed by a conceptual program logic model (Fig. 1) based on the PRECEDE-PROCEED model of behaviour change [19]. In addition to objective measurement of primary and secondary outcomes, this trial included a multi-stage evaluation of knowledge and attitudinal factors. Furthermore, a theory-informed process evaluation was used to explore how the intervention was implemented and sustained across different settings to help understand issues of program implementation, explain discrepancies between expected and observed outcomes in relation to context, and provide insights into possible causal mechanisms and effect modifiers. We hypothesised that after the intervention:

- An increased proportion of patients at high risk of recurrence would be referred for consideration of adjuvant radiotherapy or referred to the concurrent RAVES trial [Radiotherapy Adjuvant Vs Early Salvage (protocol number: TROG.08.03)] [20].
- Urologists would have increased knowledge and more positive attitudes towards the guideline recommendation.

## Methods

### Study design

**Fig. 2:**
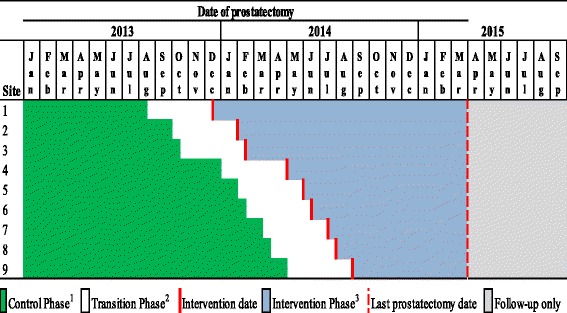
Timing of the intervention rollout in relation to date of prostatectomy ^1^Control-phase patients were those whose prostatectomy was performed between 1 January 2013 and 4 months before the CLICC intervention introductory session ^2^Transition-phase patients were those whose prostatectomy was performed between the date of the CLICC intervention introductory session and 4 months prior. This transition-phase was created because some transition patients could potentially benefit from the intervention while others could be referred or discussed before the intervention date and thus receive no such benefit ^3^Intervention-phase patients were those whose prostatectomy was performed after the CLICC intervention introductory session at the MDT to which the urologist belonged

The CLICC trial used a stepped-wedge cluster randomised design [21]. Participating Sites crossed over from the pre- to post-intervention phase in nine randomised steps, determined by a computer-generated random number sequence, with the intervention rolled out during regularly scheduled MDT meetings between 13 December 2013 and 27 August 2014 (Fig. 2).

### Study participants

#### Sites

All NSW hospitals that met the inclusion criteria of having a urological MDT and one or more members of the Urology Network within the MDT were invited.

#### Urologists

Urologists eligible for inclusion were members of a participating urological MDT, who (i) performed one or more RPs during the study period; and (ii) were a member of a participating MDT for the potential post-RP review of high-risk cases at the time the intervention commenced at that Site. The latter two eligibility criteria were specified after protocol publication [18] to enable exclusion of urologists who (i) did not perform any RPs during the study and would not contribute any clinical data; and (ii) were members of a participating MDT but presented RP patients for review at a non-participating MDT.

### Intervention alignment

In keeping with the participatory emphasis of the PRECEDE-PROCEED model [19], a prospective needs and barriers analysis, detailed in the study protocol [18], was undertaken involving consultation with multiple clinical stakeholders, consumers and representatives of cancer policy agencies to maximise engagement and to ensure that intervention elements were aligned with the local context. The needs and barriers analysis comprised: (i) two workshops with Urology Network members (*n* = 25) to identify barriers and assess feasibility within the network context; (ii) interviews with urologists (*n* = 9), clinical nurse consultants (*n* = 7) and radiation oncologists (*n* = 10) at the nine Sites to explore the membership and structure of the MDT, perceived current practice and local barriers to the implementation of the clinical practice recommendation; (iii) a nationwide survey completed by more than half of all practicing members of the Urological Society of Australia and New Zealand (USANZ) (*n* = 157) [22] to determine the extent to which barriers identified at the local level were representative of those evident in the wider urological clinical population; (iv) a network initiated focus group with consumer representatives (*n* = 15) to identify patient information priorities; (v) a 2-h meeting attended by representatives of cancer policy agencies, professional societies (including those representing urologists and radiation oncologists), urological clinical trials groups and consumer advocacy groups (*n* = 18), to evaluate identified barriers and the proposed intervention elements to address these barriers to ensure they were feasible, scalable and potentially translatable to other cancers.

Identified barriers, described fully in the study protocol [18] and summarised in Fig. 1, were considered at three levels: (i) individual clinician; (ii) patient; and (iii) hospital systems and processes, including the urological multidisciplinary team. Intervention elements were mapped to barriers using the CLICC conceptual program logic framework (Fig. 1). Through this framework, clinician level barriers (knowledge, attitudes, perceptions and norms) were mapped to physician-focused components (predisposing and reinforcing factors) and hospital level barriers (systems and processes, and culture) were mapped to context-focused components (enabling factors). Intervention elements were developed in consultation with members of the Urology Network to ensure they had face validity.

The CLICC intervention was designed to be implemented during routinely scheduled MDT meetings. Briefly, CLICC elements included the following:

#### Physician-focused components

- Peer-identified local Clinical Leaders, linked to the Urology Network and recruited by the Network Clinical Chair, plus state (Network Clinical Chair) and national (President of USANZ) opinion leaders, to reinforce key messages, model targeted referral behaviours and promote practice change (reinforcing factor)
- Non-didactic, peer-to-peer education, facilitated by the local Clinical Leader, including a video summary of the evidence underlying the clinical practice recommendation and the introduction of key messages through discussion of best clinical practice, by state and national opinion leaders, and patient experiences of care (predisposing factor)
- Dissemination of printed materials, including the full clinical practice guideline, a quick reference guide and supporting randomised controlled trial publications (predisposing factor)
- Quarterly audit and feedback reports of individual clinicians’ practice (written feedback) and study sites’ aggregated practice (written and verbal feedback by the Clinical Leader at MDT meetings) (reinforcing factor)

#### Context-focused component

- Establishment of a new ‘flagging’ process for pathology services to identify patients with high-risk features post-RP to the urological MDT coordinator for addition to the subsequent MDT meeting agenda (enabling factor).

A full description of intervention elements and how they relate to the PRECEDE-PROCEED model is provided in the study protocol [18]. Intervention tools are available upon request.

### Data collection methods

#### Primary and secondary outcomes

##### Primary outcome

The primary outcome was patient referral within 4 months after RP (‘referral’ hereafter) to either radiation oncology or to the RAVES trial [20]. The RAVES trial was designed to compare survival and quality of life outcomes for Australasian RP patients with high-risk features through randomisation to either immediate adjuvant radiotherapy or salvage radiotherapy in the event of a rise in prostate-specific antigen (PSA).

##### Secondary outcomes

Secondary outcomes were an initial patient consultation with a radiation oncologist (‘consultation’); enrolment in the RAVES trial; and commencement of radiotherapy (‘radiotherapy’), all within 6 months after RP. Enrolment in the RAVES trial could not be measured due to insufficient data recorded in patient medical records. An additional secondary outcome, discussion of the patient at a MDT meeting within 4 months after RP (‘discussion’), was added to the protocol during the trial but prior to any analysis because of the central role of MDTs in this trial.

#### Data extraction from medical records

Clinical data were extracted by independent research assistants, blinded to the date of intervention commencement, from medical records at hospitals, cancer centers and urologists’ private consulting rooms, for a minimum of 6 months after RP, using standard methods. MDT administrative records were also reviewed. Data were collected for all patients who had a RP performed by a participating urologist between 1 January 2013 and 31 March 2015, and who were subsequently found by pathology to have one or more of the three pre-specified high-risk features.

#### Explanatory factors—knowledge and attitudinal outcomes

Urologists’ knowledge, attitudes and beliefs regarding the recommendation for adjuvant radiotherapy were measured through pre- and post-intervention surveys [22, 23].

#### Process outcomes

The CLICC conceptual program logic model (Fig. 1) [18] informed the design of the process evaluation to explore how well the theory underpinning the intervention was realised in the design and delivered in the real-world context of the study to identify mechanisms of provider and organisational change. The process evaluation assessed three domains: (i) whether the intervention was implemented as intended (*implementation*); (ii) why the intervention did or did not change practice (*participation and response*); and (iii) why was or was not the intervention implemented or sustained across implementation Sites (*context*) [24]. Process outcomes were measured through document review to gather quantitative measures of intervention elements to assess implementation, participation and response, and context. Post-intervention semi-structured interviews were conducted to explore participants’ experiences of, and responses to, the intervention (predisposing, enabling and reinforcing factors) and the contextual characteristics of the nine Sites (Additional file 1: Table S1).

### Statistical methods

Patients whose RP was performed: (i) after CLICC commenced at their Site were in the intervention-phase; (ii) prior to 4 months before CLICC commenced were in the control-phase; and (iii) between CLICC commencing and 4 months prior were in the transition-phase (Fig. 1). Generalised linear regression models with Poisson distribution, log link and generalised estimating equation (GEE) adjustment for the clustering of patients within urologists were used to estimate adjusted relative proportions (RR) for patient groups (e.g. intervention, transition, control) who experienced the dichotomous study outcomes referred, discussed and consultation (radiation was not analysed in regression analyses because of referrals to the RAVES trial). RRs were obtained by exponentiation of the linear coefficients estimated by the regression models [25]. RRs were adjusted for factors listed below the relevant tables and figures. Potential modifiers of the effects of the intervention were assessed by adding terms for interaction with study phase. Whether increasing patient discussion at the MDT-level was associated with changes in patient referral was tested by creating a continuous independent variable containing the (ln) RRs corresponding to estimates of the MDT-specific intervention effects on discussion. Terms for this variable and its interaction with study phase were then added to a model with referral as the dependent variable. Exchangeable working correlation structures and robust standard errors were used in all GEE analyses.

Estimates of mean differences in pre- and post-intervention scores from Likert scale survey responses were calculated using linear regression analyses with GEE adjustment for multiple responses from the same urologists across both surveys [26].

Qualitative interviews were transcribed verbatim to produce transcripts of narrative text for thematic analysis. The CLICC evaluation framework guided the initial categorisation of text, whereby each segment of interview text was conceptually linked to one of two qualitative evaluation domains: response to the intervention (predisposing, enabling and reinforcing factors); and the contextual characteristics of the nine participating Sites. Two iterations of comparative coding were undertaken to ensure consistency.

## Results

### Participation

**Fig. 3:**
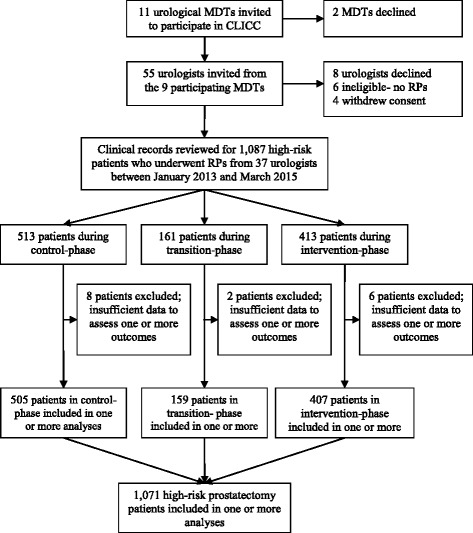
CONSORT diagram

Eleven hospitals met the inclusion criteria. Two urological MDTs declined to participate. The final sample was 37 urologists, from nine Sites, who performed RPs on 1071 patients with high-risk features during the study and for whom sufficient clinical information was available to include them in one or more analyses: 505, 159 and 407 patients in the control, transition and intervention phases, respectively (Fig. 3).

Patients’ characteristics (Table 1) were similar across study phases with the exception of regional lymph node involvement (*p* = 0·035). However, the proportions of these patients (6%) were similar in the control and intervention phases.

**Table 1:** Patients’ characteristics by study phase

Characteristic	Study phase				
	Control	Transition	Intervention	Total:	
	*n* (%)	*n* (%)	*n* (%)	*N* (%)	*p* value^
All patients:	505 (100%)	159 (100%)	407 (100%)	1071 (100%)	
Age					
Median (years)	65.0	65.0	65.0	65.0	
Quartiles (years)	59–68	58–69	61–69	60–69	
Age group					
40–59	128 (25%)	43 (27%)	81 (20%)	252 (24%)	0.145
60–69	284 (56%)	84 (53%)	231 (57%)	599 (56%)	
70+	93 (18%)	32 (20%)	95 (23%)	220 (21%)	
Extracapsular extension					
No	96 (19%)	27 (17%)	69 (17%)	192 (18%)	0.511
Yes	406 (80%)	131 (82%)	338 (83%)	875 (82%)	
Unsure	3 (1%)	1 (1%)	0 (0%)	4 (0%)	
Positive surgical margin					
No	229 (45%)	69 (43%)	198 (49%)	496 (46%)	0.087
Yes	276 (55%)	89 (56%)	204 (50%)	569 (53%)	
Unsure	0 (0%)	1 (1%)	5 (1%)	6 (1%)	
Seminal vesicle invasion					
No	395 (78%)	131 (82%)	339 (83%)	865 (81%)	0.231
Yes	109 (22%)	28 (18%)	66 (16%)	203 (19%)	
Unsure	1 (0%)	0 (0%)	2 (0%)	3 (0%)	
Regional lymph node involvement					
No	305 (60%)	98 (62%)	278 (68%)	681 (64%)	0.035
Yes	30 (6%)	5 (3%)	25 (6%)	60 (6%)	
Unsure	170 (34%)	56 (35%)	104 (26%)	330 (31%)	
Post-operative Gleason grade					
6–7	395 (78%)	133 (84%)	344 (85%)	872 (81%)	0.132
8	30 (6%)	3 (2%)	18 (4%)	51 (5%)	
9–10	77 (15%)	22 (14%)	42 (10%)	141 (13%)	
Unsure	3 (1%)	1 (1%)	3 (1%)	7 (1%)	
Number of co-morbidities					
0	103 (20%)	19 (12%)	70 (17%)	192 (18%)	0.050
1	72 (14%)	19 (12%)	45 (11%)	136 (13%)	
2	74 (15%)	23 (14%)	49 (12%)	146 (14%)	
3+	256 (51%)	98 (62%)	243 (60%)	597 (56%)	
Maximum PSA level within 4 months after RP (ng/ml)					
< 0.1	399 (79%)	137 (86%)	339 (83%)	875 (82%)	0.224
≥ 0.1	83 (16%)	16 (10%)	51 (13%)	150 (14%)	
No PSA test recorded	23 (5%)	6 (4%)	17 (4%)	46 (4%)	
Site					
Site 1	27 (5%)	14 (9%)	48 (12%)	89 (8%)	< 0.001
Site 2	11 (2%)	2 (1%)	12 (3%)	25 (2%)	
Site 3	68 (13%)	39 (25%)	120 (29%)	227 (21%)	
Site 4	51 (10%)	12 (8%)	54 (13%)	117 (11%)	
Site 5	23 (5%)	3 (2%)	19 (5%)	45 (4%)	
Site 6	77 (15%)	21 (13%)	36 (9%)	134 (13%)	
Site 7	81 (16%)	26 (16%)	34 (8%)	141 (13%)	
Site 8	120 (24%)	26 (16%)	52 (13%)	198 (18%)	
Site 9	47 (9%)	16 (10%)	32 (8%)	95 (9%)	

Data are *n* (%) unless otherwise stated, ^*p* values are for differences in % across the three groups from chi-squared tests

### Primary and secondary outcomes

#### Primary outcome—referral

**Fig. 4:**
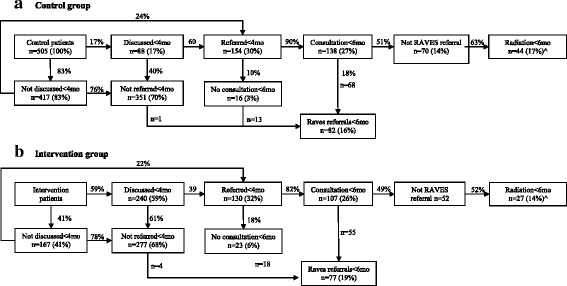
Patients’ referral pathways to radiation oncology or the RAVES trial. Percentages adjacent to connector lines represent the proportion of patients who continue from the previous category (box) into the next category. Percentages within categories (boxes) represent the proportion of all study patients, with the exception of “radiation < 6 months” where the denominator^ excludes those referred to RAVES. ^To account for RAVES referrals, the probability of radiation < 6 months is calculated as probability (consultation < 4 months) × probability(radiotherapy < 6 months|not RAVES referral) (= 27% × 63% = 17% for the control group; = 26% × 52% = 14% for the intervention group). months = months after prostatectomy

In the intervention-phase, 32% (130 of 407) of patients were referred compared with 30% (154 of 505) in the control-phase (Table 2 and Fig. 4). After adjustment for potential confounders, referral was not significantly different between the intervention and control phases (adjusted RR = 1·06; 95% CI [0.74 to 1.51]; *p* = 0·879). The effect of the intervention on referral was not significantly modified by any of the potential effect modifiers (Additional file 1: Table S2) with the exceptions of comorbidities (*p* = 0.024) and Site (*p* < 0.001).

**Table 2:** Patients’ outcomes by study phase

Characteristic	Referred^1^		Discussed^2^		Consultation^3^	
	n/N (%)	Adjusted # RR (95% CI)	n/N (%)	Adjusted # RR (95% CI)	n/N (%)	Adjusted # RR (95% CI)
All patients:	325/1071 (30%)		354/1071 (33%)		278/1071 (26%)	
Study phase						
Control	154/505 (30%)	ref.	88/505 (17%)	ref.	138/505 (27%)	ref.
Transition	41/159 (26%)	0.99 (0.68, 1.46)	26/159 (16%)	1.52 (0.90, 2.58)	33/159 (21%)	0.99 (0.72, 1.35)
Intervention	130/407 (32%)	1.06 (0.74, 1.51)	240/407 (59%)	4.32 (2.40, 7.75)	107/407 (26%)	1.05 (0.74, 1.51)
*p* value		0.879		< 0.001		0.896

^1^Patient referred within 4 months after prostatectomy to either a radiation oncologist or to the RAVES trial

^2^Patient discussed at MDT meeting within 4 months after prostatectomy

^3^Patient had consultation with radiation oncologist within 6 months after RP following referral within 4 months after RP

#Adjusted for age at prostatectomy (40–59, 60–69, 70+), extracapsular extension (no, yes, unsure), positive surgical margin (no, yes, unsure), seminal vesicle invasion (no, yes, unsure), regional lymph node involvement (no, yes, unsure), post-operative Gleason score (6–7, 8, 9–10, unsure), maximum PSA level within 4 months after RP (< 0.1 ng/ml, ≥ 0.1 ng/ml, no PSA test recorded), number of co-morbidities (0, 1, 2, 3+), site (1 through 9), calendar time period of surgery (four time periods) and urologist as the GEE clustering variable

19 patients with “unsure” extracapsular extension, positive surgical margin and/or seminal vesicle invasion were excluded from regression analysis because low numbers in those groups prevented model convergence

#### Secondary outcome—discussion

Discussion of the patient at a MDT meeting significantly increased during the intervention-phase (adjusted RR = 4.32; 95% CI [2.40 to 7.75]; *p* < 0·001) (Table 2). Fifty-nine per cent of patients during the intervention-phase (240 of 407) were discussed compared with 17% during the control-phase (88 of 505). The effect of the intervention on discussion was significantly modified by a number of patient and disease characteristics (Additional file 1: Table S3). In general, larger relative increases in the rates of discussion were observed for patients with features corresponding to lower risk of prostate cancer recurrence, such as no seminal vesicle invasion, Gleason score 6–7, or PSA ≤ 0·1 ng/ml. The effect of the intervention on discussion significantly varied by Site (*p* < 0·001) (Additional file 1: Table S3).

#### Association between MDT-level changes in patient discussion and patient referral

**Fig. 5:**
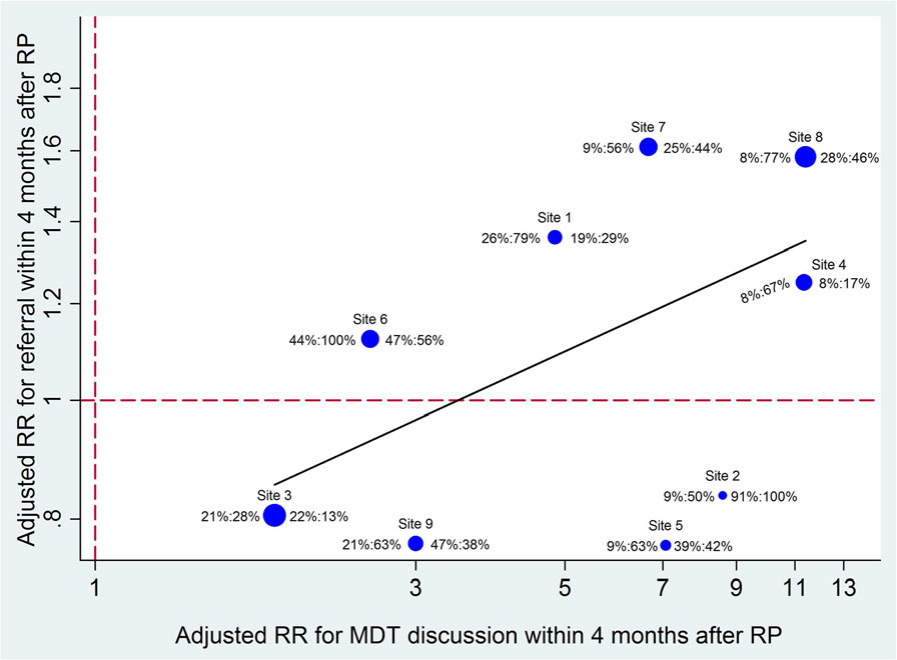
Association between changes in patient discussion at the MDT-level and changes in patient referral at the MDT-level during the intervention phase. Scatter points represent the MDT-specific RRs for referral (*y*-axis) and discussion (*x*-axis) within 4 months after prostatectomy as reported in Additional file 1: Tables S2 and S3. Numbers adjacent to the scatter points represent control:intervention percentages of patients discussed (left of scatter points) and referred (right of scatter points) within 4 months after prostatectomy. Scatter point sizes are proportional to numbers of patients. Solid line represents the predicted RRs for referral derived from regression model which effectively weights observations for Site sample size (patients); *p* = 0.001 for test of slope = 0

Sites with higher proportional increases in patients discussed tended to have higher proportional increases in referral (*p* = 0·001; Fig. 5).

The MDT recommendation was known for 217 of 240 (90%) patients who were discussed during the intervention-phase, and this was referral for 58% of these patients (140 of 240), of whom only 62 (44%) were actually referred within 4 months (Additional file 1: Table S4). Where documented in the patient’s medical record, the most common reasons for non-referral of patients with a MDT recommendation for referral were low or undetectable PSA (*n* = 45; 58%); good post-operative continence (*n* = 28; 36%); and watch and wait for salvage radiotherapy (*n* = 12; 15%) (Additional file 1: Table S5).

#### Secondary outcome—consultation

Attendance at an initial radiation oncology consultation (following referral within 4 months) was not significantly different between the intervention and control phases (intervention 26% vs control 27% adjusted RR = 1.05; 95% CI [0.74 to 1.51]; *p* = 0.896) (Table 2; potential effect modifiers shown in Additional file 1: Table S6).

#### Secondary outcome—radiotherapy

The probabilities of commencing radiotherapy were 17% and 14% in the control and intervention phases respectively) (Fig. 4).

### Knowledge and attitudinal outcomes

Twenty-nine of 37 participating urologists (78%) completed the pre-intervention survey, and 24 of 37 (65%) completed the post-intervention survey (20 [54%] completed both surveys).

Compared with pre-intervention measures, post-intervention urologists did not have increased knowledge or changed attitudes towards the guideline recommendation with one exception; there was less agreement post-intervention that the recommendation is consistent with the opinions of respected clinical colleagues (mean difference – 0.4; 95% CI [− 0.7, 0.0]; *p* = 0.027) (Additional file 1: Figure S1).

### Process outcomes

All nine Clinical Leaders (100%) and 20 of the 28 remaining participating urologists (71%) completed an end of study interview (overall response rate 78%).

#### Implementation

All Clinical Leaders and participating urologists met the minimum requirements for exposure to the intervention elements (Additional file 1: Table S7). The minimum period of exposure to CLICC intervention elements was 13 months (Site 9), and the maximum was 21 months (Site 1). Sites received a minimum of two and a maximum of four individual and aggregated quarterly feedback reports depending on date of commencement of the intervention at their Site. A total of 110 individual feedback reports and 26 Site and aggregate study level reports were distributed to participants (reports summarised in footnote to Additional file 1: Table S7). Implementation of the flagging process for identifying eligible patients to the MDT coordinator varied significantly across Sites in the proportions of patients flagged (*p* < 0·001) and in the proportions discussed amongst those flagged (*p* < 0.001) (Table 3). Public patients were significantly less likely to be flagged by pathology for discussion than private patients (RR = 0.56; 95% CI [0.42, 0.75; *p* < 0.001) (data shown in Table 3). Overall 220 of 318 flagged patients (69%) were discussed. While, as noted previously, public patients were significantly less likely to be flagged for discussion than private patients, there was no significant difference in the proportion discussed among those flagged (RR = 1.15; 95% CI [0.89, 1.49]; *p* = 0.282). Three Sites (Sites 2, 3 and 8) adapted the process for adding patients to the MDT agenda after receiving notification of eligible patients from pathology. At Site 2, the MDT coordinator did not list eligible patients on the agenda for discussion unless a request was received from the participating urologist. At Sites 3 and 8 discussion of patients was delayed until after receipt of the first post-operative PSA test result.

**Table 3:** Integration of the MDT flagging process into routine care (ranked by percent of patients discussed among those flagged)

		Flagged			Discussed^1^ among those flagged	
Characteristic	N1^	n1 (% of N1)	Adjusted # RR (95% CI)	N2^^	n2 (% of N2)	Adjusted # RR (95% CI)
All patients:	407	318 (78%)		318	220 (69%)	
Hospital						
Site 6	36	34 (94%)	1.13 (1.03, 1.25)	34	34 (100%)	3.30 (2.70, 4.03)
Site 5	19	6 (32%)	0.46 (0.16, 1.32)	6	6 (100%)	3.14 (2.50, 3.95)
Site 1	48	32 (67%)	0.94 (0.81, 1.09)	32	30 (94%)	2.94 (2.29, 3.78)
Site 4	54	40 (74%)	0.96 (0.78, 1.17)	40	36 (90%)	2.92 (2.29, 3.72)
Site 8	52	48 (92%)	1.13 (1.07, 1.21)	48	40 (83%)	2.74 (2.23, 3.37)
Site 2	12	8 (67%)	0.79 (0.75, 0.84)	8	6 (75%)	2.47 (2.03, 3.02)
Site 7	34	25 (74%)	0.94 (0.67, 1.33)	25	18 (72%)	2.37 (1.59, 3.54)
Site 9	32	29 (91%)	1.42 (1.24, 1.63)	29	20 (69%)	2.09 (1.29, 3.37)
Site 3	120	96 (80%)	ref.	96	30 (31%)	ref.
*p* value			< 0.001			< 0.001
Insurance						
Private	329	280 (85%)	ref.	280	190 (68%)	ref.
Public	78	38 (49%)	0.56 (0.42, 0.75)	38	30 (79%)	1.15 (0.89, 1.49)
*p* value			< 0.001			0.282

^Intervention group patients

^^Intervention group patients who were flagged

^1^Patient discussed at MDT meeting within 4 months after prostatectomy

#Adjusted for site and insurance with urologist as the GEE clustering variable

#### Response

Identification of eligible cases by the pathologist to the MDT coordinator, through the flagging process, was considered by the majority of interviewees to be the most essential and sustainable element in achieving practice change (21 of 29; 72%). The automatic nature of the process, requiring no action on the part of the urologist, was noted as a key facilitator in uptake. “[Flagging of high-risk cases was] most important especially for high volume cancer centres where it is easy to provide excellent care but patients still fall through the cracks due to sheer numbers. The MDT list was manageable because the patients flagged are the right ones that should be given priority over others.” [Clinical Leader—Site 6] Some, however, did not find the flagging helpful because of the timing: “two weeks after the operation there is no progress, no six-week PSA and continence status is not known so you don’t have a feel if radiotherapy is appropriate, necessary or a hindrance.” [PU—Site 8].

Feedback reports were noted to be helpful by nearly half (14 of 29; 48%), and more than a third (37%) considered the provision of feedback to be the main benefit of participation in the trial allowing comparisons between Sites to see how practice and surgical outcomes varied. “Individual reporting to the urologists enables them to see their own results – some were surprised by their low referral rates. I’m not sure the overall pattern data made much difference because there were only one or two funny outliers. Personal information is more useful.” [Clinical Leader—Site 7].

There was a mixed response to other intervention components, namely, printed educational materials and the role of the Clinical Leader.

Seven interviewees (24%) found the printed educational materials useful with four of these highlighting them as the most helpful element of the trial. “[Printed materials] were very clear about the way forward for the management of these patients.” [PU—Site 5] However, one participant noted: “This information has been around for a while but there are problems with the results so I guess that’s why we need to think about it.” [PU—Site  3].

While 5 of the 29 interviewees (17%) found the CLICC introductory video helpful: “Flagging followed by the video – it was concise, pitched at the right level and did all the things a good educational video should.” [CL—Site 4], others considered it impersonal and the content too lay: “Clinical content was too simple. If you are attending conferences and up to date with Continuing Professional Development then you would know about adjuvant radiotherapy.” [PU—Site 4].

Of the 20 urologist participant interviewees, four (20%) (Sites 4, 5, 7 and 8) noted the influence of the Clinical Leader as important in achieving desired outcomes but none articulated a reason for this. Of note, only three of the nine Clinical Leaders (from Sites 5, 6 and 7) viewed their role as one of an opinion leader to actively influence and promote behaviour change, and two (from Sites 1 and 4) expressed that they did not perceive it as their role to influence colleagues or offer support to change practice: “Didn’t see my role was to tell my colleagues to follow the guideline and I didn’t do it.” [CL—Site 1].

#### Context

RP caseload varied (Table 1), as did the frequency, organisation and record-keeping of MDT meetings, all of which impacted upon implementation of the flagging process and the proportion of flagged patients that were discussed. All Sites had a designated MDT coordinator (administrator or nurse) responsible for scheduling and agendas, with the exception of Site 3 where organisation was delegated to the incumbent urology registrars. This Site had the highest RP caseload (Table 1) and also discussed the lowest proportion of patients amongst those flagged (Table 3). High-patient volume, insufficient logistical planning for implementation of the flagging process and lack of support from the Clinical Leader were all identified as issues at this Site. Across all four Sites where the Clinical Leaders noted issues with implementation of the MDT flagging process these related to resourcing for public pathology services (Sites 1, 5, 6 and 7). Only two Sites were able to achieve similar rates of public patients flagged as private patients. Both of these Sites had higher public patient volume and had a lead pathologist that took responsibility for flagging and reporting on public patients at the MDT.

### Sensitivity analyses

Sensitivity analyses showed that results were robust to a variety of different assumptions and/or statistical methods (Additional file 1: Figures S2 and S3).

## Discussion

The CLICC trial used mixed methods to assess clinicians’ knowledge and attitudinal outcomes alongside behavioural outcomes measured through independent medical record review. A process evaluation, underpinned by behavioural constructs hypothesised a priori, was conducted in parallel to identify mechanisms of provider and organisational change, aid interpretation of outcomes, and increase the utility of findings for broader implementation science theory and practice.

The trial did not result in a significant increase in the primary outcome of referral. This is consistent with the results of the corresponding participant surveys indicating a lack of change in self-reported knowledge and attitudes including treatment preference, and continued challenges to underpinning evidence for the benefit of adjuvant radiotherapy, by some, in post-intervention interviews. Nevertheless, there was evidence that the CLICC intervention was more effective in changing referral in some Sites than others. Following implementation of a new ‘flagging’ process, there was a greater than threefold proportional increase in the secondary outcome of patient discussion at a MDT meeting with 59% being discussed during the intervention-phase compared with 17% during the control-phase. Sites with the largest increases in discussion also tended to have greater increases in referral. Of note, the four Sites that had the highest proportional increases in referral (Sites 1, 4, 7 and 8) were amongst the five Sites with the highest proportional increases in patients discussed at a MDT meeting. This is consistent with the hypothesis that introducing new systems or processes, tailored to prospectively identified barriers, can enable desired behaviour change if they are integrated and adopted into routine clinical practice as designed. To the best of our knowledge, this is a novel trial of such a ‘flagging’ process to standardise selection criteria for presentation of cases to MDTs across multiple clinical sites.

The CLICC intervention was implemented with fidelity. There was a high level of clinician participation with more than three quarters of eligible invitees participating. The non-participation of two eligible sites was a potential limitation to the generalisability of the intervention. While there was 100% participation at five of nine Sites, not all eligible urologists participated at all Sites which may have resulted in volunteer bias. Patients who were identified as potentially eligible for inclusion (2378 low-risk and high-risk patients) represented nearly half (47%) of the 5017 Medicare reimbursed RPs claimed in NSW during the study period 1 January 2013 to 31 March 2015 [27]. This implies the results are likely to be representative of wider clinical practice. It is acknowledged, however, that the intervention effect on the primary and two of the three secondary outcomes, varied significantly by Site so variation in effectiveness is likely to be evident more widely.

Within the CLICC conceptual program logic model, flagging of eligible cases by the pathologist to the MDT coordinator for discussion at a MDT meeting was hypothesised to enable referral by overcoming clinician level barriers associated with variable engagement with, and selective presentation of cases to, the MDT. Following discussion by the MDT, however, overall less than half of intervention-phase patients with a MDT recommendation for referral were actually referred within the recommended timeframe. The lack of actual referral for patients with a MDT recommendation for referral demonstrates that altered practice to discuss more patients did not inevitably lead to referral behaviour in line with the MDT recommendation. This is likely due, at least in part, to the persisting attitudes of urologists challenging the benefit of adjuvant radiotherapy. However, it may also be due to urologists’ perceptions of the role of MDTs. The establishment of MDTs in cancer care has been advocated widely internationally [28] and nationally [29] including the introduction in 2006 of two Australian Commonwealth Government Medicare Benefit Scheme (MBS) payment items [30] to support clinicians participating in cancer case conferences. Our data show that fewer than 20% of patients with high-risk prostate cancer were discussed by a MDT prior to the implementation of the flagging process. While the rate of discussion significantly increased as a direct result of the intervention, our findings raise questions about the function and utility of MDT discussion in cancer care. Lack of referral may have been due to irregularities in MDT record-keeping, which resulted in inconsistent communication of recommendations to the consulting clinician. Whether recommendations were known or not, follow-up care remained at the discretion of the consulting clinician. This trial demonstrates that implementation of new enabling systems or processes may be necessary but not sufficient to bring about change in clinical practice that is reliant on an individual clinician’s behaviour. It has been noted by others that interventions focused on systemic or structural changes are generally more successful than interventions focused on individual factors (e.g. attitudes). Possible solutions in the current context could be the introduction of standardised recording of MDT recommendations in patients’ medical records to ensure they are accessible at the point of care, or the implementation of a direct care pathway, for example, through a letter sent to the patient’s general practitioner. There is arguably a need to review how MDTs operate more generally.

The proportion of patients who attended an initial consultation with a radiation oncologist did not significantly change. The overall probability of commencing radiotherapy in our sample was 15%—marginally higher than another recent estimate from 37 hospitals in Victoria, Australia [11]. In part, low rates of adjuvant radiotherapy are due to low rates of referral. However, among the 122 intervention- and control-phase (non-RAVES) patients who attended an initial consultation, only a little over half commenced radiotherapy. Further, the probability of commencing radiotherapy decreased slightly between the control and intervention phases. This is consistent with retrospective analyses of data from nearly 100,000 patients in the US National Cancer Data Base showing a significant *decline* in the use of post-RP radiotherapy from 9.1 to 7.3% between 2005 and 2011. Locally, the results of surveys of members of the Urological Society of Australia and New Zealand (USANZ) conducted in 2012 and 2015, demonstrate urologists not participating in the CLICC trial were significantly less favourable towards adjuvant radiotherapy in 2015 than in 2012 [22, 23]. This is, perhaps, due to the development of biomarkers and the recent availability of genomic classifier tests which, if applied routinely, may offer a more individualised approach to the post-prostatectomy management of men with high-risk features [31, 32]. These findings highlight the intricacy of implementing behaviour change interventions within the context of a shifting external landscape.

The lack of significant change in our primary outcome is consistent with other ‘real-world’ implementation trials, which have noted the complexities and challenges associated with the translation of evidence into clinical practice [33]. Further research is necessary to explore the reasons for heterogeneity of CLICC intervention effectiveness between Sites. It should be noted that the CLICC trial was primarily conceptualised as a clinician-focused intervention with the specific aim of changing provider referral behaviours. Consequently, ethical approvals did not permit direct patient interaction. Future research could examine whether a patient-oriented intervention can effect change in clinical practice.

## Conclusions

Overall, this trial provokes the conclusion that whilst guidelines can make recommendations about best care, changing practice routinely across the health system remains a seemingly intractable challenge and supports the view that interventions that enable and reinforce system-level change are likely to have greater effect than interventions that seek to predispose individual-level change. In conjunction, interventions focused on system-level changes need to be complemented by processes to ensure treatment recommendations are acted upon at the point of care. Finally, the results support the ongoing focus on how cancer care can become more multidisciplinary and enable informed choice for patients.

## Additional file

Additional file 1: **Table S1.** Process evaluation domains and data collection methods. **Table S2.** Potential effect modifiers of the intervention effect on prevalence of referral to radiation oncologist or RAVES within 4 months after prostatectomy. **Table S3.** Potential effect modifiers of the intervention effect on prevalence of patients being discussed at a MDT meeting within 4 months after prostatectomy. **Table S4.** MDT recommendations within 4 months after prostatectomy by referral status during the intervention phase. **Table S5.** Reasons for non-referral as recorded in urologist notes among the 78 intervention group cases with a MDT recommendation for referral who were not referred within 4 months of prostatectomy. **Table S6.** Potential effect modifiers of the intervention effect on prevalence of an initial consultation with a radiation oncologist within 6 months after prostatectomy. **Table S7.** Site-level exposures to CLICC intervention elements. **Figure S1.** Comparisons between pre-intervention and post-intervention responses—attitudes towards recommendation that ‘patients with extracapsular extension, seminal vesicle involvement or positive surgical margins receive post-operative external beam radiation therapy within four months of surgery’. **Figure S2.** Sensitivity analyses for outcomes of referred and discussed. **Figure S3.** Sensitivity analyses for outcomes of consultation and radiotherapy. (DOCX 87 kb)
