# Supplementary material for: A multidisciplinary team-oriented intervention to increase guideline recommended care for high-risk prostate cancer: A stepped-wedge cluster randomised implementation trial
Source: Implement Sci. 2018 Mar 12;13:43. doi: 10.1186/s13012-018-0733-x (PMC5848547; doi:10.1186/s13012-018-0733-x)
Supplement: Supplementary file 1 — Table S1. Process evaluation domains and data collection methods. Table S2. Potential effect modifiers of the intervention effect on prevalence of referral to radiation oncologist or RAVES within 4 months after prostatectomy. Table S3. Potential effect modifiers of the intervention effect on prevalence of patients being discussed at a MDT meeting within 4 months after prostatectomy. Table S4. MDT recommendations within 4 months after prostatectomy by referral status during the intervention phase. Table S5. Reasons for non-referral as recorded in urologist notes among the 78 intervention group cases with a MDT recommendation for referral who were not referred within 4 months of prostatectomy. Table S6. Potential effect modifiers of the intervention effect on prevalence of an initial consultation with a radiation oncologist within 6 months after prostatectomy. Table S7. Site-level exposures to CLICC intervention elements. Figure S1. Comparisons between pre-intervention and post-intervention responses—attitudes towards recommendation that ‘patients with extracapsular extension, seminal vesicle involvement or positive surgical margins receive post-operative external beam radiation therapy within four months of surgery’. Figure S2. Sensitivity analyses for outcomes of referred and discussed. Figure S3. Sensitivity analyses for outcomes of consultation and radiotherapy. (DOCX 87 kb) [file 13012_2018_733_MOESM1_ESM.docx]

**Table S1: Process evaluation domains and data collection methods**

| **Process evaluation domain** | **Definition** | **Data collection methods** |
| --- | --- | --- |
| ***Implementation*** | Implementation was considered as the extent to which the intervention was implemented as planned with fidelity, the degree to which essential elements were delivered, the level of exposure, and local adaptation. | - Participant fidelity checklist |
| ***Participation*** and  ***Response*** | Participation was considered in terms of recruitment and reach, specifically: the proportion of the target population that actually received the intervention, and their representativeness.  Response was considered as the extent to which multidisciplinary teams integrated and adopted new knowledge, systems and processes into their routine practice. Unintended consequences and outcomes in response to the intervention were also evaluated. | - Recruitment database: overall number of urologists at each Site; the number who consented, declined, did not respond or withdrew from the study - Medicare claims data - Date of commencement of the intervention - Attendance of urologists at the intervention session - Aggregate level of exposure to, and adaptation of, intervention elements at each Site - Extent to which multidisciplinary teams integrated the MDT identification process into routine practice: date the patient was identified by pathology; whether the patient was added to a MDT agenda; date of discussion; and MDT recommendation (where known) |
| ***Context*** | Context was documented to enable consideration of any setting characteristics that may have influenced the delivery of the intervention or impacted on its effectiveness or maintenance/sustainability across Sites to facilitate interpretation of outcomes and maximise the potential for scale up and spread. | - Frequency, organisation and record keeping of MDT meetings - Provision of public and private pathology services. - Patient volume, public/private case mix and other setting characteristics (from medical record review) |

**Table S2: Potential effect modifiers of the intervention effect on prevalence of referral to radiation oncologist or RAVES within 4 months after prostatectomy**

|  | **Referred^1^/Total (%)** | |  |  |
| --- | --- | --- | --- | --- |
| **Potential effect modifier** | **Control** | **Intervention** | **Adjusted RR# for intervention effect  (95%CI)** | ***p-value* for interaction** |
| **All patients:** | 154/505 (30%) | 130/407 (32%) | 1.06 (0.74, 1.51)^^ | n/a |
| **Age group** |  |  |  |  |
| 40-59 | 36/128 (28%) | 28/81 (35%) | 1.26 (0.82, 1.96) | 0.195 |
| 60-69 | 96/284 (34%) | 78/231 (34%) | 0.94 (0.63, 1.40) |  |
| 70+ | 22/93 (24%) | 24/95 (25%) | 1.18 (0.81, 1.72) |  |
| **Extracapsular extension** | |  |  |  |
| No | 22/96 (23%) | 15/69 (22%) | 0.86 (0.53, 1.38) | 0.169 |
| Yes | 131/406 (32%) | 115/338 (34%) | 1.11 (0.76, 1.60) |  |
| Unsure | 1/3 (33%) | 0/0 (.) | n/a^ |  |
| **Positive surgical margin** |  |  |  |  |
| No | 64/229 (28%) | 54/198 (27%) | 1.02 (0.73, 1.44) | 0.730 |
| Yes | 90/276 (33%) | 76/204 (37%) | 1.08 (0.71, 1.63) |  |
| Unsure | 0/0 (.) | 0/5 (0%) | n/a^ |  |
| **Seminal vesicle invasion** |  |  |  |  |
| No | 93/395 (24%) | 88/339 (26%) | 1.04 (0.72, 1.51) | 0.487 |
| Yes | 61/109 (56%) | 41/66 (62%) | 1.16 (0.77, 1.73) |  |
| Unsure | 0/1 (0%) | 1/2 (50%) | n/a^ |  |
| **Regional lymph node involvement** | |  |  |  |
| No | 94/305 (31%) | 87/278 (31%) | 1.09 (0.76, 1.55) | 0.875 |
| Yes | 19/30 (63%) | 14/25 (56%) | 1.19 (0.67, 2.13) |  |
| Unsure | 41/170 (24%) | 29/104 (28%) | 1.01 (0.65, 1.56) |  |
| **Post-operative Gleason score** |  |  |  |  |
| 6-7 | 109/395 (28%) | 103/344 (30%) | 1.09 (0.76, 1.58) | 0.492 |
| 8 | 8/30 (27%) | 3/18 (17%) | 0.67 (0.32, 1.39) |  |
| 9-10 | 35/77 (45%) | 23/42 (55%) | 1.03 (0.60, 1.78) |  |
| Unsure | 2/3 (67%) | 1/3 (33%) | n/a^ |  |
| **Number of co-morbidities** |  |  |  |  |
| 0 | 32/103 (31%) | 19/70 (27%) | 0.73 (0.51, 1.05) | 0.024 |
| 1 | 29/72 (40%) | 19/45 (42%) | 1.22 (0.78, 1.90) |  |
| 2 | 17/74 (23%) | 17/49 (35%) | 1.58 (0.89, 2.82) |  |
| 3+ | 76/256 (30%) | 75/243 (31%) | 1.06 (0.71, 1.57) |  |
| **Maximum PSA level within 4 months after RP (ng/ml)** | | |  |  |
| < 0.1 | 103/399 (26%) | 99/339 (29%) | 1.13 (0.79, 1.62) | 0.443 |
| ≥0.1 | 44/83 (53%) | 26/51 (51%) | 0.90 (0.54, 1.49) |  |
| No PSA test recorded | 7/23 (30%) | 5/17 (29%) | 0.92 (0.38, 2.24) |  |
| **Hospital** |  |  |  |  |
| Site 1 | 5/27 (19%) | 14/48 (29%) | 1.36 (0.43, 4.30) | <0.001 |
| Site 2 | 10/11 (91%) | 12/12 (100%) | 0.84 (0.56, 1.25) |  |
| Site 3 | 15/68 (22%) | 16/120 (13%) | 0.81 (0.58, 1.11) |  |
| Site 4 | 4/51 (8%) | 9/54 (17%) | 1.25 (0.75, 2.08) |  |
| Site 5 | 9/23 (39%) | 8/19 (42%) | 0.76 (0.35, 1.66) |  |
| Site 6 | 36/77 (47%) | 20/36 (56%) | 1.12 (0.81, 1.55) |  |
| Site 7 | 20/81 (25%) | 15/34 (44%) | 1.61 (0.83, 3.12) |  |
| Site 8 | 33/120 (28%) | 24/52 (46%) | 1.58 (1.03, 2.44) |  |
| Site 9 | 22/47 (47%) | 12/32 (38%) | 0.76 (0.47, 1.24) |  |

^1^Patient referred within 4 months after prostatectomy to either a radiation oncologist or to the RAVES trial.

# Adjusted for age at prostatectomy (40-59, 60-69, 70+), extracapsular extension (No, Yes, Unsure), positive surgical margin (No, Yes, Unsure), seminal vesicle invasion (No, Yes, Unsure), regional lymph node involvement (No, Yes, Unsure), post-operative Gleason score (6-7, 8, 9-10, Unsure), maximum PSA level within 4 months after RP (<0.1 ng/ml, ≥ 0.1ng/ml, no PSA test recorded), number of co-morbidities (0, 1, 2, 3+), Site (1 through 9), calendar time period of surgery (4 time periods) and urologist as the GEE clustering variable.

^^ Results from original analyses repeated here for convenience

^ Convergence not achieved due to low group numbers

**Table S3: Potential effect modifiers of the intervention effect on prevalence of patients being discussed at a MDT meeting within 4 months after prostatectomy**

|  | **Discussed^1^/Total (%)** | |  |  |
| --- | --- | --- | --- | --- |
| **Potential effect modifier** | **Control** | **Intervention** | **Adjusted RR# for intervention effect  (95%CI)** | ***p-value* for effect modification** |
| **All patients:** | 88/505 (17%) | 240/407 (59%) | 4.32 (2.40, 7.75)^^ | n/a |
| **Age group** |  |  |  |  |
| 40-59 | 23/128 (18%) | 50/81 (62%) | 4.07 (2.25, 7.36) | 0.284 |
| 60-69 | 45/284 (16%) | 136/231 (59%) | 4.81 (2.45, 9.43) |  |
| 70+ | 20/93 (22%) | 54/95 (57%) | 3.62 (1.93, 6.78) |  |
| **Extracapsular extension** |  |  |  |  |
| No | 13/96 (14%) | 35/69 (51%) | 4.79 (2.39, 9.62) | 0.644 |
| Yes | 75/406 (18%) | 205/338 (61%) | 4.23 (2.33, 7.69) |  |
| Unsure | 0/3 (0%) | 0/0 (.) | n/a^ |  |
| **Positive surgical margin** |  |  |  |  |
| No | 39/229 (17%) | 115/198 (58%) | 4.69 (2.30, 9.58) | 0.567 |
| Yes | 49/276 (18%) | 122/204 (60%) | 4.08 (2.31, 7.22) |  |
| Unsure | 0/0 (.) | 3/5 (60%) | n/a^ |  |
| **Seminal vesicle invasion** |  |  |  |  |
| No | 59/395 (15%) | 199/339 (59%) | 5.02 (2.68, 9.40) | 0.038 |
| Yes | 29/109 (27%) | 40/66 (61%) | 2.91 (1.47, 5.76) |  |
| Unsure | 0/1 (0%) | 1/2 (50%) | n/a^ |  |
| **Regional lymph node involvement** | |  |  |  |
| No | 53/305 (17%) | 154/278 (55%) | 3.88 (2.03, 7.42) | <0.001 |
| Yes | 18/30 (60%) | 12/25 (48%) | 1.05 (0.51, 2.19) |  |
| Unsure | 17/170 (10%) | 74/104 (71%) | 7.90 (4.15, 15.00) |  |
| **Post-operative Gleason score** |  |  |  |  |
| 6-7 | 58/395 (15%) | 205/344 (60%) | 4.85 (2.54, 9.27) | 0.016 |
| 8 | 5/30 (17%) | 11/18 (61%) | 4.37 (1.87, 10.21) |  |
| 9-10 | 24/77 (31%) | 21/42 (50%) | 2.20 (1.16, 4.16) |  |
| Unsure | 1/3 (33%) | 3/3 (100%) | n/a^ |  |
| **Number of co-morbidities** |  |  |  |  |
| 0 | 15/103 (15%) | 37/70 (53%) | 4.47 (2.54, 7.86) | 0.291 |
| 1 | 14/72 (19%) | 27/45 (60%) | 3.19 (1.50, 6.77) |  |
| 2 | 14/74 (19%) | 29/49 (59%) | 3.75 (1.78, 7.90) |  |
| 3+ | 45/256 (18%) | 147/243 (60%) | 4.82 (2.61, 8.89) |  |
| **Maximum PSA level within 4 months after RP (ng/ml)** | | |  |  |
| < 0.1 | 58/399 (15%) | 204/339 (60%) | 5.03 (2.64, 9.61) | <0.001 |
| ≥0.1 | 25/83 (30%) | 29/51 (57%) | 2.50 (1.45, 4.30) |  |
| No PSA test recorded | 5/23 (22%) | 7/17 (41%) | 2.44 (1.16, 5.13) |  |
| **Hospital** |  |  |  |  |
| Site 1 | 7/27 (26%) | 38/48 (79%) | 4.84 (2.03, 11.49) | <0.001 |
| Site 2 | 1/11 (9%) | 6/12 (50%) | 8.59 (0.90, 82.01) |  |
| Site 3 | 14/68 (21%) | 33/120 (28%) | 1.85 (0.78, 4.37) |  |
| Site 4 | 4/51 (8%) | 36/54 (67%) | 11.35 (3.64, 35.44) |  |
| Site 5 | 2/23 (9%) | 12/19 (63%) | 7.07 (2.72, 18.34) |  |
| Site 6 | 34/77 (44%) | 36/36 (100%) | 2.57 (1.24, 5.30) |  |
| Site 7 | 7/81 (9%) | 19/34 (56%) | 6.66 (3.19, 13.94) |  |
| Site 8 | 9/120 (8%) | 40/52 (77%) | 11.41 (6.52, 19.97) |  |
| Site 9 | 10/47 (21%) | 20/32 (63%) | 3.00 (1.30, 6.93) |  |

^1^Patient discussed at MDT meeting within 4 months after prostatectomy.

# Adjusted for age at prostatectomy (40-59, 60-69, 70+), extracapsular extension (No, Yes, Unsure), positive surgical margin (No, Yes, Unsure), seminal vesicle invasion (No, Yes, Unsure), regional lymph node involvement (No, Yes, Unsure), post-operative Gleason score (6-7, 8, 9-10, Unsure), maximum PSA level within 4 months after RP (<0.1 ng/ml, ≥ 0.1ng/ml, no PSA test recorded), number of co-morbidities (0, 1, 2, 3+), Site (1 through 9), calendar time period of surgery (4 time periods) and urologist as the GEE clustering variable.

^^ Results from original analyses repeated here for convenience

^ Convergence not achieved due to low group numbers

**Table S4: MDT recommendations within 4 months after prostatectomy by referral status during the intervention phase**

|  |  | **Actual referral** | | | | |
| --- | --- | --- | --- | --- | --- | --- |
|  |  | **Referred within 4 months after RP^1^** |  | **Referred within 6 months after RP^2^** |  | **Referred by end of study follow-up^3^** |
| **MDT recommendation within 4 months after RP** | **N** | **n (%)** |  | **n (%)** |  | **n (%)** |
| Referral to RT or RAVES | 140 | 62 (44%) |  | 67 (48%) |  | 72 (51%) |
| Watch and wait | 42 | 6 (14%) |  | 8 (19%) |  | 8 (19%) |
| Other recommendation | 35 | 14 (40%) |  | 14 (40%) |  | 15 (43%) |
| Recommendation not recorded | 23 | 12 (52%) |  | 12 (52%) |  | 12 (52%) |
| Case not discussed within 4 months after RP | 167 | 36 (22%) |  | 47 (28%) |  | 53 (32%) |
| **TOTAL (all patients during intervention phase):** | **407** | **130 (32%)** |  | **148 (36%)** |  | **160 (39%)** |

^1^Patient referred within 4 months after prostatectomy to either a radiation oncologist or the RAVES trial

^2^Patient referred within 6 months after prostatectomy to either a radiation oncologist or the RAVES trial

^3^Patient referred by end of study follow-up to either a radiation oncologist or the RAVES trial (median follow-up time of 11.4 months in intervention phase)

**Table S5: Reasons for non-referral as recorded in urologists’ notes among the 78 intervention group cases with a MDT recommendation for referral who were not referred within 4 months of prostatectomy**

| **Possible reasons recorded ^** | **# of responses** | **% of n=78**  **non-referred cases** |
| --- | --- | --- |
| PSA low or undetectable | 45 | 58% |
| Continence is good | 28 | 36% |
| Watch and wait for salvage | 12 | 15% |
| Continence is bad | 4 | 5% |
| Patient preference | 0 | 0% |
| Other | 4 | 5% |
| No reason recorded in notes | 25 | 32% |

**^ Urologists may have recorded more than one reason for any one case**

**Table S6 Potential effect modifiers of the intervention effect on prevalence of an initial consultation with a radiation oncologist within 6 months after prostatectomy**

|  | **Consultation^1^/Total (%)** | |  |  |
| --- | --- | --- | --- | --- |
| **Potential effect modifier** | **Control** | **Intervention** | **Adjusted RR# for intervention effect  (95%CI)** | ***p-value* for interaction** |
| **All patients:** | 138/505 (27%) | 107/407 (26%) | 1.05 (0.74, 1.51)^^ | n/a |
| **Age group** |  |  |  |  |
| 40-59 | 34/128 (27%) | 23/81 (28%) | 1.20 (0.80, 1.80) | 0.420 |
| 60-69 | 84/284 (30%) | 66/231 (29%) | 0.97 (0.67, 1.42) |  |
| 70+ | 20/93 (22%) | 18/95 (19%) | 1.10 (0.71, 1.69) |  |
| **Extracapsular extension** | |  |  |  |
| No | 22/96 (23%) | 13/69 (19%) | 0.82 (0.52, 1.30) | 0.128 |
| Yes | 115/406 (28%) | 94/338 (28%) | 1.10 (0.75, 1.62) |  |
| Unsure | 1/3 (33%) | 0/0 (.) | n/a^ |  |
| **Positive surgical margin** |  |  |  |  |
| No | 57/229 (25%) | 39/198 (20%) | 0.91 (0.61, 1.35) | 0.137 |
| Yes | 81/276 (29%) | 68/204 (33%) | 1.15 (0.78, 1.69) |  |
| Unsure | 0/0 (.) | 0/5 (0%) | n/a^ |  |
| **Seminal vesicle invasion** |  |  |  |  |
| No | 81/395 (21%) | 69/339 (20%) | 1.03 (0.69, 1.53) | 0.301 |
| Yes | 57/109 (52%) | 37/66 (56%) | 1.20 (0.81, 1.77) |  |
| Unsure | 0/1 (0%) | 1/2 (50%) | n/a^ |  |
| **Regional lymph node involvement** | |  |  |  |
| No | 86/305 (28%) | 69/278 (25%) | 1.06 (0.72, 1.56) | 0.768 |
| Yes | 19/30 (63%) | 13/25 (52%) | 1.27 (0.69, 2.33) |  |
| Unsure | 33/170 (19%) | 25/104 (24%) | 1.12 (0.73, 1.74) |  |
| **Post-operative Gleason score** |  |  |  |  |
| 6-7 | 98/395 (25%) | 82/344 (24%) | 1.07 (0.73, 1.56) | 0.132 |
| 8 | 7/30 (23%) | 2/18 (11%) | 0.60 (0.29, 1.22) |  |
| 9-10 | 32/77 (42%) | 22/42 (52%) | 1.21 (0.73, 2.02) |  |
| Unsure | 1/3 (33%) | 1/3 (33%) | n/a^ |  |
| **Number of co-morbidities** |  |  |  |  |
| 0 | 30/103 (29%) | 15/70 (21%) | 0.65 (0.43, 0.97) | <0.001 |
| 1 | 25/72 (35%) | 17/45 (38%) | 1.49 (1.00, 2.21) |  |
| 2 | 14/74 (19%) | 15/49 (31%) | 1.79 (1.04, 3.09) |  |
| 3+ | 69/256 (27%) | 60/243 (25%) | 1.04 (0.68, 1.61) |  |
| **Maximum PSA level within 4 months after RP (ng/ml)** | | |  |  |
| < 0.1 | 90/399 (23%) | 76/339 (22%) | 1.08 (0.74, 1.57) | 0.955 |
| ≥0.1 | 42/83 (51%) | 26/51 (51%) | 1.01 (0.62, 1.65) |  |
| No PSA test recorded | 6/23 (26%) | 5/17 (29%) | 1.18 (0.49, 2.80) |  |
| **Hospital** |  |  |  |  |
| Site 1 | 5/27 (19%) | 13/48 (27%) | 1.36 (0.45, 4.11) | 0.323 |
| Site 2 | 10/11 (91%) | 11/12 (92%) | 0.74 (0.47, 1.15) |  |
| Site 3 | 12/68 (18%) | 12/120 (10%) | 0.87 (0.51, 1.48) |  |
| Site 4 | 4/51 (8%) | 9/54 (17%) | 1.51 (0.89, 2.56) |  |
| Site 5 | 8/23 (35%) | 8/19 (42%) | 0.93 (0.39, 2.22) |  |
| Site 6 | 29/77 (38%) | 13/36 (36%) | 1.08 (0.80, 1.46) |  |
| Site 7 | 20/81 (25%) | 12/34 (35%) | 1.46 (0.58, 3.65) |  |
| Site 8 | 30/120 (25%) | 17/52 (33%) | 1.19 (0.56, 2.53) |  |
| Site 9 | 20/47 (43%) | 12/32 (38%) | 0.95 (0.58, 1.55) |  |

^1^ Patient had consultation with radiation oncologist within 6 months after prostatectomy following referral within 4 months after prostatectomy.

# Adjusted for age at prostatectomy (40-59, 60-69, 70+), extracapsular extension (No, Yes, Unsure), positive surgical margin (No, Yes, Unsure), seminal vesicle invasion (No, Yes, Unsure), regional lymph node involvement (No, Yes, Unsure), post-operative Gleason score (6-7, 8, 9-10, Unsure), maximum PSA level within 4 months after RP (<0.1 ng/ml, ≥ 0.1ng/ml, no PSA test recorded), number of co-morbidities (0, 1, 2, 3+), Site (1 through 9), calendar time period of surgery (4 time periods) and urologist as the GEE clustering variable.

^^ Results from original analyses repeated here for convenience

^ Convergence not achieved due to low group numbers


**Table S7: Site level exposures to CLICC intervention elements**

|  | Opinion Leaders | | | Provider Education and Printed Materials | | | | Audit & Feedback^ | | | | Automated Systems | |
| --- | --- | --- | --- | --- | --- | --- | --- | --- | --- | --- | --- | --- | --- |
|  | Clinical Leader | Urology Network Co-Chair* | President of USANZ* | CLICC Video | Full CPG** | RCT*** papers | CLICC printed resource | Report 1 | Report 2 | Report 3 | Report 4 | Public pathology MDT identification | Private pathology MDT identification |
| Site 1 | ✓ | ✓ | ✓ | ✓ | ✓ | ✓ | ✓ | ✓ | ✓ | ✓ | ✓ | ✓ | ✓ |
| Site 2 | ✓ | ✓ | ✓ | ✓ | ✓ | ✓ | ✓ | ✓ | ✓ | ✓ | ✓ | ✓ | ✓ |
| Site 3 | ✓ | ✓ | ✓ | ✓ | ✓ | ✓ | ✓ | ✓ | ✓ | ✓ | ✓ | ✓ | ✓ |
| Site 4 | ✓ | ✓ | ✓ | ✓ | ✓ | ✓ | ✓ | ✓ | ✓ | ✓ |  | ✓ | ✓ |
| Site 5 | ✓ | ✓ | ✓ | ✓ | ✓ | ✓ | ✓ | ✓ | ✓ | ✓ |  | ✓ | ✓ |
| Site 6 | ✓ | ✓ | ✓ | ✓ | ✓ | ✓ | ✓ | ✓ | ✓ |  |  | ✓ | ✓ |
| Site 7 | ✓ | ✓ | ✓ | ✓ | ✓ | ✓ | ✓ | ✓ | ✓ |  |  |  | ✓ |
| Site 8 | ✓ | ✓ | ✓ | ✓ | ✓ | ✓ | ✓ | ✓ | ✓ |  |  | ✓ | ✓ |
| Site 9 | ✓ | ✓ | ✓ | ✓ | ✓ | ✓ | ✓ | ✓ | ✓ |  |  | ✓ | ✓ |

* CLICC video

** CPG: Australian Cancer Network Clinical Practice Guideline for the Management of Locally Advanced and Metastatic Prostate Cancer[14]

*** Randomised controlled trial

^ Feedback reports:

Feedback Report 1: individual, site level and aggregate study level pre-CLICC (pre-intervention) outcome data (1 January 2013 – end of month prior to CLICC intervention commencement)

Feedback Report 2: site level and aggregate study level pre-CLICC (pre-intervention) outcome data / individual and site level post-CLICC MDT discussion data

Feedback Report 3: individual, site level and aggregate study level pre-CLICC (pre-intervention) and post-CLICC outcome data / individual and site level post-CLICC MDT discussion data

Feedback Report 4: individual, site level and aggregate study level post-CLICC MDT discussion data / aggregate study level pre-CLICC (pre-intervention) outcome data

**Figure S1: Comparisons between pre-intervention and post-intervention responses - attitudes towards recommendation that ‘*patients with extracapsular extension, seminal vesicle involvement or positive surgical margins receive post-operative external beam radiation therapy within four months of surgery*’**

**This recommendation:*

^ Scores correspond to a 5-point Likert type scale with scoring 1=Strongly disagree, 2=Disagree, 3=Neither agree nor disagree, 4=Agree, 5=Strongly agree; “Don’t know” and missing responses were excluded from analyses

Survey #1 = Pre-intervention Survey #2 = Post-intervention

*Full survey questions are available from corresponding author. Some items were reverse coded for analyses and these are reflected in question labels.

**Figure S2: Sensitivity analyses for outcomes of referred and discussed**

^a^ Patient referred within 4 months after prostatectomy to either a radiation oncologist or to the RAVES trial

^b^ Patient discussed at MDT meeting within 4 months after prostatectomy

# All analyses with the exception of #4 were adjusted for age at prostatectomy (40-59, 60-69, 70+), extracapsular extension (No, Yes, Unsure), positive surgical margin (No, Yes, Unsure), seminal vesicle invasion (No, Yes, Unsure), regional lymph node involvement (No, Yes, Unsure), post-operative Gleason score (6-7, 8, 9-10, Unsure), maximum PSA level within 4 months after RP (<0.1 ng/ml, ≥ 0.1ng/ml, no PSA test recorded), number of co-morbidities (0, 1, 2, 3+), Site (1 through 9), calendar time period of surgery (4 time periods) In addition: GEE analyses (ie analyses #1-6 and #8) included cohort Site as a fixed effect and urologist as the panel variable; the linear mixed model analysis (ie analyses #7) included random effect terms for Site and urologists nested within Sites.

^ Results from original analyses repeated here for convenience

(1) Excludes patients who were referred to radiation oncologist before their prostatectomy

(2) For each of the 2 outcomes above, respectively, patients were excluded if they were referred and/or discussed within 4 months after prostatectomy but their urologist recorded the reason as salvage therapy, or no specific reason was recorded but they had a PSA reading of ≥0.1 within 4 months after prostatectomy

(3) Excludes patients who did not have a post-surgical consultation within 4 months after prostatectomy

(4) Adjusted only for calendar time period of surgery (4 time periods), age at prostatectomy (40-59, 60-69, 70+), and Study Site (1 through 9), with urologist defined as the panel variable.

(5) Excludes patients of the urologist with highest case-load comprising 13.7% of all prostatectomies in the study

(6) Excludes patients from the Site with highest case-load among Sites comprising 20.9% of all prostatectomies in the study.

(7) Results from a linear mixed model analyses with random effect terms for Site and urologists nested within Sites.

(8) The two outcomes of referred and discussed were assessed at 6 months rather than 4 months.

**Figure S3: Sensitivity analyses for outcomes of consultation**

^c^ Patient had consultation with radiation oncologist within 6 months after prostatectomy following referral within 4 months after prostatectomy

# All analyses with the exception of #4 were adjusted for age at prostatectomy (40-59, 60-69, 70+), extracapsular extension (No, Yes, Unsure), positive surgical margin (No, Yes, Unsure), seminal vesicle invasion (No, Yes, Unsure), regional lymph node involvement (No, Yes, Unsure), post-operative Gleason score (6-7, 8, 9-10, Unsure), maximum PSA level within 4 months after RP (<0.1 ng/ml, ≥ 0.1ng/ml, no PSA test recorded), number of co-morbidities (0, 1, 2, 3+), Site (1 through 9), calendar time period of surgery (4 time periods). In addition: GEE analyses (ie analyses #1-6) included cohort Site as a fixed effect and urologist as the panel variable; the linear mixed model analysis (ie analyses #7) included random effect terms for Site and urologists nested within Sites.

^ Results from original analyses repeated here for convenience

(1) Excludes patients who were referred to radiation oncologist before their prostatectomy

(2) Patients were excluded if they had a consultation within 6 months after prostatectomy but their urologist recorded the reason as salvage therapy, or no specific reason was recorded but they had a PSA reading of ≥0.1 within 6 months after prostatectomy

(3) Excludes patients who did not have a post-surgical consultation within 4 months after prostatectomy

(4) Adjusted only for calendar time period of surgery (4 time periods), age at prostatectomy (40-59, 60-69, 70+), and Site (1 through 9), with urologist defined as the panel variable.

(5) Excludes patients of the urologist with highest case-load comprising 13.7% of all prostatectomies in the study

(6) Excludes patients from the Site with highest case-load among Sites comprising 20.9% of all prostatectomies in the study.

(7) Results from a linear mixed model analyses with random effect terms for Site and urologists nested within Sites.
